# Supplementary material for: Telementoring versus face-to-face mentoring in the training of scleral fixation surgery of intraocular lenses
Source: PLoS One. 2023 Dec 28;18(12):e0290040. doi: 10.1371/journal.pone.0290040 (PMC10754431; doi:10.1371/journal.pone.0290040)
Supplement: S1 Table — (DOCX) [file pone.0290040.s001.docx]

SUPPLEMENTARY MATERIAL

RUBRIC

**GLOBAL SCORE SCALE FOR OBJECTIVE STRUCTURED ASSESSMENT OF TECHNICAL SKILL**

Please rate the candidate’s performance on the following scale (complete by two independent surgeons after watching edited surgical videos blindly)

**SCORES:**

1 - “performed inappropriately or inefficiently to perform the surgical step”

2 - “performed with some hesitation, with additional maneuvers, but in a satisfactory manner”

3 – “performed well and without hesitation, showing respect for technique, tissues, time and mobility”

**SUPPLEMENTARY TABLE 1.** The evaluation form used in the study containing nine surgical steps graded on a 3-point Likert scale.

**SUCCESS RATE**

Surgical success is achieved when the student can complete the procedure with proper fixation of the IOL in the model. Please judge at the end of the procedure on the success or failure of mentoring by following the guidelines below (completed by the mentor in the end of each procedure):

If the mentor marks **one of the** criteria below, the mentoring is classified as a failure:

- Failure in the transmission of images and/or audio, which makes it impossible to complete the mentoring.
- Surgeon was not able to complete one of the surgical steps.
- The procedure had any surgical complication such as loss of suture, damage to the intraocular lens, displacement of the lens into the vitreous cavity.

If none of the above items were checked, please rate the procedure as successful.

- FAILURE
- SUCCESS
