# Supplementary material for: Telementoring versus face-to-face mentoring in the training of scleral fixation surgery of intraocular lenses
Source: PLoS One. 2023 Dec 28;18(12):e0290040. doi: 10.1371/journal.pone.0290040 (PMC10754431; doi:10.1371/journal.pone.0290040)
Supplement: Attachment — Submitted filename: Response to Reviewers.docx [file pone.0290040.s002.docx]

**Response to Reviewers:**

| Reviewer Number | Original comments of the reviewer | Reply by the author(s) | Changes done on page number |
| --- | --- | --- | --- |
| Journal Requirements: | 1. Please ensure that your manuscript meets PLOS ONE's style requirements, including those for file naming.  2. Please state what role the funders took in the study.  3. We note that Figure 1 in your submission contain copyrighted images. We require you to either (1) present written permission from the copyright holder to publish these figures specifically under the CC BY 4.0 license, or (2) remove the figures from your submission: | We thank the Editorial Office and reviewers for the comments and suggestions.  We have addressed the concerns raised, and we now believe that the manuscript is substantially improved. We hope that you will agree and find that the manuscript is now suitable for publication.   1) Ok. Done. 2) Ok. Done. 3) We have recreated an image to replace the previous "Figure 1", without copyrights. | This change can be found highlighted at:   1) -- 2) Page 14, Line 323 and 324. 3) -- |
| Reviewer #1 | 1) I am still unclear about the telementoring workflow - so was video being transmitted or only images? This is an important point to clarify 2) Is career time post-training? 3) Results section: please clarify this statement ' There is a lack of evidence between the groups (p-values > 0.05), and the standardized effects sizes were inferior to 0.2, indicating a very small effect under the Cohen’s d scale cutoffs. 4) Given the surgical success scale used, is there any reference point to actual OR success? This would help contextualize the 98.5% and 95.5% values. 5) If not inexperience with NGENUITY, why do you suppose did those with 13+ years of experience underperform wtih telemonitoring? | 1) Yes, telementoring took place via videoconference. To avoid misinterpretation by readers, we have replaced the word "teleconferencing" with "videoconferencing" in the methods section. 2) We considered the baseline of the career time as the year in which the participant entered the medical residency in ophthalmology. 3) Here is written as “lack of evidence” because your findings refer to a situation where the analysis does not provide sufficient evidence to support the hypothesis (e.g., the difference between the two arms on the outcome had a p-value superior to the commonly adopted significance level [0.05]). It means that the observed results are inconclusive or do not provide strong support for a specific conclusion. It is common to observe that when RCTs describe group differences that are not statistically significant, authors mistakenly state that p-values > 0.05 as the intervention having no effect. No effect means evidence of absence which is different from lack of absence. The difference between *lack of evidence* and *evidence of absence* is classically described and explored in the statistical note written by Altman & Bland (1995). In our work, we had a special attention to clearly describing such statements. Now, in the manuscript, it might be found as follows on page 7 and 8, lines 170-175. 4) In fact, surgical success was considered if the participant managed to reach the end of the procedure without committing any criterion considered as failure. They are: “Failure in the transmission of images and/or audio, which makes it impossible to complete the mentoring”; “surgeon was not able to complete one of the surgical steps” and “the procedure had any surgical complication such as loss of suture, damage to the IOL, or displacement of the IOL into the vitreous cavity”. This information is already contained in the manuscript. 5) We do not know for sure, nor did we find clear reasons for this data obtained. At first, we thought that this subgroup possibly had less contact with the Ngenuity® system, but as mentioned in the manuscript, this did not happen: "Inexperience with the Ngenuity® system is probably not the cause for these results since in our sample, 73% of this subgroup had previously used this technology." | This change can be found highlighted at:   1) Page 5, Line 99. 2) – 3) Page 7 and 8, Lines 170-175. 4) Page 5 and 6, Lines 119-124. 5) -- |
| Reviewer #2 | 1) In Table 2, in the p-values row, "0.000" should be replaced with "p<0.001". | 1) Ok. Done. | This change can be found highlighted at:   1) Table 2. |
